# A Gold Nanocage Probe Targeting Survivin for the Diagnosis of Pancreatic Cancer

**DOI:** 10.3390/pharmaceutics15051547

**Published:** 2023-05-19

**Authors:** Lina Song, Shuai Ren, Yali Yue, Ying Tian, Zhongqiu Wang

**Affiliations:** Department of Radiology, Jiangsu Province Hospital of Chinese Medicine, Affiliated Hospital of Nanjing University of Chinese Medicine, Nanjing 210029, China; shuairen@njucm.edu.cn (S.R.);

**Keywords:** Au nanocage, pancreatic ductal adenocarcinoma, targeting nanoprobe, survivin-targeting imaging

## Abstract

In this paper, Au nanocages (AuNCs) loaded with the MRI contrast agent gadolinium (Gd) and capped with the tumor-targeting gene survivin (Sur–AuNC•Gd–Cy7 nanoprobes) were designed and applied as a targeted imaging agent for pancreatic cancer. With its capacity to transport fluorescent dyes and MR imaging agents, the gold cage is an outstanding platform. Furthermore, it has the potential to transport different drugs in the future, making it a unique carrier platform. The utilization of Sur–AuNC•Gd–Cy7 nanoprobes has proven to be an effective means of targeting and localizing survivin-positive BxPC-3 cells within their cytoplasm. By targeting survivin, an antiapoptotic gene, the Sur–AuNC•Gd–Cy7 nanoprobe was able to induce pro-apoptotic effects in BxPC-3 pancreatic cancer cells. The biocompatibility of AuNCs•Gd, AuNCs•Gd–Cy7 nanoparticles, and Sur–AuNC•Gd–Cy7 nanoprobes is evaluated through the hemolysis rate assay. The stability of AuNCs•Gd, AuNCs•Gd–Cy7 nanoparticles, and Sur–AuNC•Gd–Cy7 nanoprobes was evaluated by determining their hydrodynamic dimensions following storage in different pH solutions for a corresponding duration. Excellent biocompatibility and stability of the Sur–AuNC•Gd–Cy7 nanoprobes will facilitate their further utilization in vivo and in vitro. The surface-bound survivin plays a role in facilitating the Sur–AuNC•Gd–Cy7 nanoprobes’ ability to locate the BxPC-3 tumor. The probe was modified to incorporate Gd and Cy7, thereby enabling the simultaneous utilization of magnetic resonance imaging (MRI) and fluorescence imaging (FI) techniques. In vivo, the Sur–AuNC•Gd–Cy7 nanoprobes were found to effectively target and localize survivin-positive BxPC-3 tumors through the use of MRI and FI. After being injected via the caudal vein, the Sur–AuNC•Gd–Cy7 nanoprobes were found to accumulate effectively in an in situ pancreatic cancer model within 24 h. Furthermore, these nanoprobes were observed to be eliminated from the body through the kidneys within 72 h after a single injection. This characteristic is crucial for a diagnostic agent. Based on the aforementioned outcomes, the Sur–AuNC•Gd–Cy7 nanoprobes have significant potential advantages for the theranostic treatment of pancreatic cancer. This nanoprobe possesses distinctive characteristics, such as advanced imaging abilities and specific drug delivery, which offer the possibility of enhancing the precision of diagnosis and efficacy of treatment for this destructive illness.

## 1. Introduction

The incidence of pancreatic ductal adenocarcinoma (PDAC), a deadly disease, has increased twofold worldwide over the past two decades. In 1990, 196,000 patients were diagnosed with pancreatic cancer, whereas in 2017, the number increased to 441,000 [1]. Cancer centers in the United States have reported a consistent annual increase of 1% in pancreatic cancer cases, with expectations of it becoming the second leading cause of cancer-related deaths in the country by 2030 [2]. As the age structure of the population changes, China is also expected to face this predicament in the future, according to the development index.

The poor prognosis of pancreatic cancer is largely due to the challenges of early detection. In fact, more than 90% of tumors are diagnosed at an advanced stage, when they have already spread beyond the pancreas, with systemic metastasis observed in over half of them (52%) [3]. This leaves little room for a positive outcome. Therefore, it is imperative to prioritize early diagnosis and treatment of PDAC to truly enhance the overall treatment efficacy of pancreatic cancer [1,3]. This approach can gradually tackle the clinical problem of high mortality rates associated with pancreatic cancer.

The development of pancreatic cancer is characterized by an ongoing and fluctuating process that involves the aberrant expression of various biomolecules, including CA199, CEA, the P53 gene, the P16 gene, the urokinase-type plasminogen activator receptor (uPAR), mucin (muc), survivin, and plectin-1 [4,5]. According to certain academics, the survivin gene is infrequently expressed in healthy tissues, whereas it is prominently expressed in embryonic tissues and cancerous tumors such as pancreatic, breast, and lung cancer [6]. In accordance with studies, the expression of the survivin gene is negligible in normal pancreatic tissues and chronic pancreatitis lesions but is notably elevated in approximately 75% of pancreatic cancer tissues [7]. The implication is that survivin holds promise as an exceptionally targeted objective for attaining early targeted diagnosis and accurate treatment of pancreatic cancer. In a prior investigation undertaken by our team, we succeeded in creating a nanoprobe that displays exceptional T2 imaging characteristics for pancreatic cancer [8], with a particular emphasis on survivin. These results suggest that survivin represents a feasible target for further exploration.

In recent times, the use of gold nanomaterials has brought about remarkable developments in the field of tumor diagnosis and treatment [9,10,11]. Because of their visibility and ease of functionalization, AuNPs are considered a promising approach for anticancer therapy. In vitro and preclinical studies using various AuNP-based conjugates are underway to deliver commonly used chemotherapeutic drugs such as docetaxel (DTX) and 5-fluorouracil [12]. The outstanding biocompatibility, ease of surface modification, and surface plasmon resonance characteristics are all significant features of gold nanomaterials. The findings of a study [13] indicate that the use of gold nanoclusters to assist in the delivery of siRNA targeting NGF (GNC–siRNA) is a highly effective approach for silencing the NGF gene and treating pancreatic cancer. Through its potent ability to downregulate NGF expression in Panc-1 cells and pancreatic tumors, the GNC–siRNA complex has proven to be an effective inhibitor of tumor progression in three different pancreatic tumor models (including subcutaneous, orthotopic, and patient-derived xenograft models), all without any negative side effects.

Additionally, gold nanocages possess hollow and porous configurations, endowing them with a broad spectrum of light scattering and absorption as well as a considerable cross-sectional area of light absorption. This renders them useful for a variety of optical imaging modalities such as photoacoustic imaging, dark field imaging, and optical coherence tomography [14]. Liu et al. have introduced a new nanoplatform that employs gold nanorods for photothermal therapy in the NIR-II range, combined with inhibition of N6-methyladenosine (m6A) demethylase, to enhance photothermal immunotherapy against prostate cancer. The research also offers an understanding of how m6A RNA methylation and PTT of AuNRs can work together to create a hopeful strategy for cancer immunotherapy [15]. Zhu and colleagues suggest that adopting a biomimetic approach to the synthesis of gold nanomaterials can have multiple benefits. Not only does it increase the efficiency of gold utilization and reduce waste, but it also results in materials that are less toxic to living organisms and more biocompatible. These advantages make biomimetic gold nanomaterials an attractive option for a range of applications, including bioanalysis, early cancer detection, cell imaging, and tumor therapy [16]. Li and colleagues have conducted a thorough analysis of the latest developments in stimuli-responsive gold nanocages (AuNCs) for cancer diagnosis and treatment. They have underscored the potential of AuNCs in the context of stimuli-responsive drug delivery systems (DDSs), while also pointing out the possible clinical translation of AuNCs, as well as their limitations, for cancer diagnosis and treatment [17]. The MR

I contrast agent of choice is typically gadolinium (Gd), which functions to decrease the T1 relaxation time of water protons in the tissue under the influence of an external magnetic field. This leads to an improved signal disparity between the lesion and surrounding tissue, ultimately enhancing tissue contrast.

**Scheme 1:**
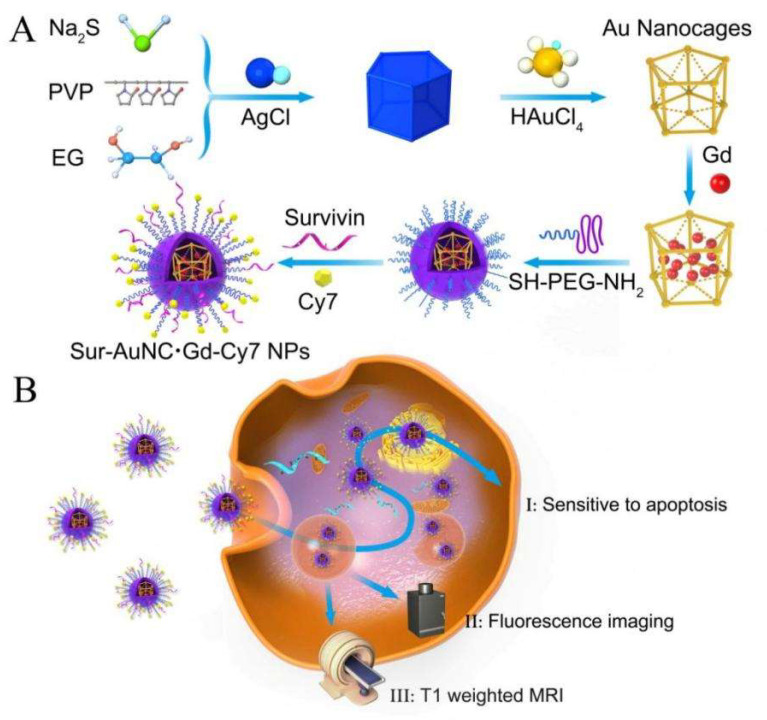
(**A**) Schematic illustration of the preparation of Sur–AuNC·Gd–Cy7 nanoprobes. (**B**) Schematic illustration of the tumor-responsive nanoprobes Sur–AuNC·Gd–Cy7 for promoting cell apoptosis and fluorescence/MR imaging.

In order to create a new nanoplatform that integrates the aforementioned functions, we have devised a novel type of nanoprobe that utilizes Au nanocages and is actively targeted (known as Sur–AuNC∙Gd–Cy7). These nanoprobes are designed for use in multimodal imaging diagnosis, combining both fluorescence and magnetic resonance imaging techniques (Scheme 1). The creation of this multifunctional diagnosis nanoprobe involved the incorporation of Survivin into PEG-coated Au nanocages and the introduction of Gd through electrostatic interaction within the interior. Subsequently, the Au nanocages that had been functionalized were united with the fluorescent dye Cy7 by means of electrostatic interactions. The findings of our investigation demonstrate that the Sur–AuNC∙Gd–Cy7 nanoprobes exhibit commendable multi-modal imaging proficiency for pancreatic cancer, both in vitro and in vivo. Our study has thus furnished a significant imaging framework for identifying innovative diagnostic agents for pancreatic cancer.

## 2. Materials and Methods

### 2.1. Materials

All the reagents used in the experiment were purchased from commercial companies and were used without extra purification. Chloroauric acids (HAuCl_4_), polyvinyl pyrrolidone (PVP), dimethyl formamide (DMF), and other agents used in this experiment were purchased from Sinopharm Chemical Reagent Co. Ltd. (Shanghai, China). The compounds: 1, 4, 7, 10-tetraazododecane, 1, 4, 7, 10-tetracarboxylic acid, and gadolinium complex (DOTA–Gd–NHS ester) came from Xi’an ruixi Biological Technology Co., Ltd. (Xi’an, China) Sulfo-N-hydroxysuccinimide (sulfo–NHS) and Cy7–NHS were purchased from Sigma Aldrich (St. Louis, MO, USA). Thiol poly-(ethylene glycol) amine (SH–PEG–NH_2_) was purchased from Beijing Jenkem Technology Co., Ltd. (Beijing, China). The compound, 1-ethyl-(3-dimethylaminopropyl) carbodiimide hydrochloride (EDC–HCl), was purchased from J&K Chemicals. Survivin’s antisense nucleotide was from Shanghai Sangon Biotech Co., Ltd. (Shanghai, China).

Bovine serum albumin (BSA) was obtained from Solarbio (Beijing, China). Dimethyl sulfoxide (DMSO) was purchased from Sangon Biological Engineering Technology & Services Co., Ltd. (Shanghai, China). Phosphate Buffer Saline (PBS buffer) was from Thermo Fisher Scientific (Waltham, MA, USA). Annexin V-FITC and CCK-8 kits were purchased from Beyotime Biotechnology (Shanghai, China). Deionized water, which was purified by the Millipore Water Purification System (Burlington, MA, USA), was used throughout the study.

### 2.2. Synthesis of Au Nanocage (AuNC) and PEG Modified Gd Loading AuNC (PEG–AuNC·Gd) Nanoprobes

The Au nanocages (AuNC) were prepared via a replacement reaction. PVP aqueous solution (30 mL, 1 mg/mL) was heated to 90 °C in a conical flask. A prefabricated cubic silver nanoparticle (1 mL) solution was added to the hot solution with a stirring rate of 260–340 rpm at the same temperature. After 1 min, the solution of HAuCl_4_ (0.1 mM) was dropped into the reaction system at a rate of 0.75 mL/min. The color of the reaction solution changed from red to purple and then turned to light blue. At this point, the total solution of HAuCl_4_ added to the system was about 6 mL.

The reaction system was cooled to room temperature after continued stirring for two minutes. The reaction solution was washed with a saturated NaCl solution to remove silver ions. Then, the sample was centrifuged and washed with distilled water several times to remove superfluous PVP and NaCl. The centrifugal speed was 8500 rpm, and the time was 15 min. At last, the reaction product (AuNC) was collected and redispersed into distilled water. The AuNC aqueous solution was stored at 4 °C for future use.

By adding an aqueous solution of HCl (0.02 M), the pH of a 40-milliliter AuNC solution in water was lowered to 5.0. After adding DOTA–Gd–NHS ester solution (10 mg/mL, 0.2 mL), the mixture of AuNC and Gd solution was oscillated at room temperature for 1 h. The reaction product (AuNC·Gd) was purified by centrifuging (8000 rpm, 10 min, 3 times). SH–PEG–NH_2_ (5 mg) was dissolved in carbonate buffer (20 mL, pH = 8), which was mixed with sodium dodecyl sulfate (SDS) solution (0.1%, 1 mL) and AuNC·Gd aqueous solution. At room temperature, the mixture was subjected to oscillation for a duration of 12 h. Then the reaction product (PEG–AuNC·Gd) was collected by centrifugation and stored at 4 °C for future use.

### 2.3. Synthesis of Cy7-Coated AuNC·Gd (PEG–AuNC·Gd–Cy7) Nanoprobes and Survivin Targeting Nanoprobe Sur–AuNC·Gd–Cy7

Survivin antisense nucleotide (8.6 nmol) was dissolved in diluted hydrochloric acid (200 μL, pH = 5) and mixed with EDC (50 μL, 10 mg/mL) and NHS solution (60 μL, 10 mg/mL). After oscillating for 30 min, they were added to the PEG–AuNC·Gd aqueous solution with Cy7–NHS (10 μL, 10 mg/mL). The mixture was subjected to oscillation for the entire night at room temperature while being kept away from light. Then the reaction product (Sur–AuNC·Gd–Cy7) was collected by centrifuging and re-suspended in carbonate buffer with SDS (0.1%).

The synthesis of AuNC·Gd–Cy7 was the same as above without adding the survivin antisense nucleotide.

### 2.4. Characterization of AuNC Nanocages and Sur–AuNC·Gd–Cy7 Nanoprobes

The size and morphology of AuNC nanocages were characterized by Transmission electron microscopy (TEM) and high-resolution TEM (HRTEM) (JEOL-2100, Tokyo, Japan). Once the solution of AuNC–PEG was diluted, it was carefully applied in drops of 10 μL to copper mesh covered with a carbon film (200 mesh). The treated mesh was then allowed to air dry naturally in a cool, dry place. Using a transmission electron microscope (TEM), the morphology of the copper mesh was later analyzed and photographed.

In order to test the solutions of AuNC•Gd, AuNC•Gd–Cy7, and Sur–AuNC•Gd–cy7, they will first be diluted 100 times in pure water using a volumetric bottle. The diluted samples will then be transferred to a sample tube, and their concentrations will be measured using Inductively Coupled Plasma (ICP-AES, Optima 5300DV, PerkinElmer, Waltham, MA, USA).

Using an ultrasonic probe, the AuNC•Gd, AuNC•Gd–Cy7, and Sur–AuNC•Gd–Cy7 solutions were dispersed, and samples were placed in a cuvette. The hydrodynamic size and zeta potential of the nanoprobe were subsequently determined using a particle size analyzer (Malvern Zetasizer, Worcestershire, UK).

Different concentrations (0, 0.625, 1.25, 2.5, 5, and 10 mg/mL) of prepared AuNC•Gd, AuNC•Gd–Cy7, and Sur–AuNC•Gd–Cy7 solutions were used to measure their magnetic resonance imaging capability. The measurements were taken using a 3.0T magnetic resonance with TR = 1200 ms, TE = 100 ms, FOV = 100 × 120 mm, and slice thickness = 3 mm. The longitudinal relaxation rate was calculated based on the longitudinal relaxation time. After dispersing AuNC•Gd, AuNC•Gd–Cy7, and Sur–AuNC•Gd–Cy7 solutions with an ultrasonic probe, samples were placed in a cuvette. The ultraviolet spectrogram of AuNC∙Gd, AuNC•Gd–Cy7, and Sur–AuNC∙Gd–Cy7 nanoprobes was tested on a UV-visible spectrophotometer (UV-3600, Shimadzu, Kyoto, Japan).

The Sur–AuNC∙Gd–Cy7 nanoprobes (0.5 mM) were placed in solutions with different pH (5, 6, 7, 8, 9, 10), and then all of the solutions were left at room temperature for a month to study the colloidal stability of Sur–AuNC∙Gd–Cy7 nanoprobes.

### 2.5. Cell Culture

The pancreatic cancer cell lines (PANC-1 and BxPC-3) were originally obtained from the Type Culture Collection of the Chinese Academy of Sciences (Shanghai, China). Cells were cultured in RPMI-1640 medium (BxPC-3) or DMEM medium (PANC-1) supplemented with 10% fetal bovine serum (FBS), 100 U/mL penicillin, and 100 U/mL streptomycin at 37 °C in a humidified 5% CO_2_ atmosphere. The human pancreatic duct epithelial cell line HTERTHPNE was from Yuanduan Bio Co. (Nanjing, China) and cultured in RPMI-1640 medium. Cells in the exponential phase were selected for the experiments.

The culture medium was replaced every two days, and the growth of cells was observed in real-time. Cells from the 3rd to 10th generations were in a more favorable state. After being digested with trypsin (0.25%), the cells were centrifuged, collected, and re-suspended in a culture medium at a concentration of about 1 × 10^4^/mL. The cell suspension was added to 96-well plates (100 μL/well) or 6-well plates (2 mL/well). After about 6 to 12 h, cells were attached to the wall for future use.

### 2.6. Survivin Expression in Pancreatic Cancer Cell Lines

Western blotting was utilized to test the survivin expression in pancreatic cancer cell lines (BxPC-3 and PANC-1) and human pancreatic duct epithelial cells (HTERT-HPNE). After treatment, the cell lysates of all groups were collected, and the protein content was measured. The primary antibody survivin (16 KD) was used in this study. Cells were harvested while they were in the logarithmic phase, washed three times with PBS, and lysed in lysis buffer (1% PMSF to protect cell proteins). The cell protein concentration was determined by a protein assay kit. The same amount of lysate (6 μg) was separated on SDS-PAGE gels and transferred onto a polyvinylidenedifluoride (PVDF) membrane. The membrane was blocked with tris-buffered saline (TBS) containing 0.1% tween 20 and 5% nonfat milk at room temperature for 2 h. Then, they were incubated with the primary antibody, survivin (1:1000) overnight at 4 °C. After washing, the membranes were probed with horseradish peroxidase (HRP)-labeled goat anti-mice IgG at 1:300 dilutions and subsequently stained with the mixture of ECLA and EDLB (1:1). Anti-GADPH was used as a control to ensure equal loading. The blots were identified by chemiluminescence.

### 2.7. In Vitro TEM Imaging of Cells Culturing with Sur–AuNC∙Gd–Cy7 Nanoprobes

TEM was employed in vitro to visually detect the intracellular distribution of Sur–AuNC·Gd–Cy7 nanoprobes. After culturing with the Sur–AuNC∙Gd–Cy7 nanoprobe for 24 h, BxPC-3 cells and HTERT-HPNE cells were collected by washing, digesting, and centrifuging. The cell clusters were fixed with 2.5% glutaraldehyde overnight. Then the cell clusters were treated with osmium trioxide (1%, for 1.5 h), a group of ethanol at different concentrations (20%, 30%, 40%, 50%, 60%, 70%, 90%, and 100%, 10 min each), and uranyl acetate (2%, for 1 h) in turn. Next, cell clusters were dehydrated in ethanol (100%) for 1 h and soaked in propylene oxide (twice for 15 min each). Then they were mixed with propylene oxide and araldite resin (1:1) overnight at room temperature. At last, cell clusters were infiltrated with fresh araldite resin three times (3 h to 4 h each) and embedded with araldite resin at 60 °C for 48 h. Finally, the prepared cell clusters were sliced into super thin sections and stained with uranyl acetate (1%) and lead citrate (0.2%) to observe the targeting effects of the Sur–AuNC∙Gd–Cy7 nanoprobe with TEM.

### 2.8. In Vitro Cell MR Imaging and Fluorescence Imaging

Sur–AuNC·Gd–Cy7 nanoprobes were employed to investigate the imaging impact on cells using in vitro magnetic resonance imaging and fluorescence imaging techniques.

After exposure to different concentrations of AuNC∙Gd nanoparticles and Sur–AuNC∙Gd–Cy7 nanoprobes for 24 h, cells were collected. The agarose solution (1%) was selected as a better carrier for the immobilization of cells and made sure the cells were distributed uniformly. Then they were placed on a 3.0T MR scanner to observe the T1 signals using a head and neck coil. The sequence parameter was as follows: TR = 490 ms, TE = 14 ms, FOV = 230 mm × 230 mm, matrix size = 256 × 256, slice thickness = 1 mm. To establish a blank control, untreated cells were utilized, and a negative control was set up using distilled water.

After exposure to different concentrations of Sur–AuNC∙Gd–Cy7 nanoprobe for 24 h, BxPC-3 cells were collected. The cell suspensions were placed on a small animal vivisection imaging system to observe the fluorescence signals. Distilled water was utilized as a negative control, while the untreated cells served as a blank control.

### 2.9. Cell Cytotoxicity Experiments

Cytotoxicity of AuNC∙Gd and Sur–AuNC∙Gd–Cy7 nanoprobes on pancreatic cancer cell lines was analyzed by the MTT kit and Annexin V-FITC/PI double staining assay. As above, cells were seeded in a 96-well plate (5 × 10^3^/well, 100 μL/well). After cells were attached to the wall, different concentrations of AuNC∙Gd and Sur–AuNC∙Gd–Cy7 nanoprobe (0, 0.1562, 0.3125, 0.625, 1.25, 2.5, 5, 10 µg [Au]/mL) were added to the culture system. The culture system was placed in an incubator for 24 h. Then 20 μL MTT was added to each well of the 96-well plate and incubated at 37 °C for 4 h. The supernatant was sucked out carefully, and the formazan, which was the reaction product of MTT and live cells, was re-suspended in 150 μL of dimethyl sulfoxide (DMSO). The absorbance was measured using a microplate reader (BIO-RAD, model 680) at 490 nm. The cells treated with different concentrations of AuNC∙Gd and Sur–AuNC∙Gd–Cy7 nanoprobe were the experimental groups (Read A). The same concentration of AuNC∙Gd and Sur–AuNC∙Gd–Cy7 nanoprobes with culture medium were used as condition controls (Read B). The negative control was cells untreated with AuNC∙Gd or Sur–AuNC∙Gd–Cy7 nanoprobe (Read C). The blank control was only medium (read D). The cell viability was calculated as follows: cell viability (%) = (A − B)/(C − D) × 100%.

Pancreatic cancer cells were cultured with AuNC∙Gd (5, 10 µg [Au]/mL) and Sur–AuNC∙Gd–Cy7 (5, 10 µg [Au]/mL) nanoprobes in 6-well plates for 24 h. The cells were harvested immediately after being washed with PBS and digested with trypsin (out of EDTA). Annexin V-FITC and PI staining were performed according to the manufacturer’s recommended conditions. The apoptosis was analyzed on a flow cytometer (Calibur, BD, New York, NY, USA).

### 2.10. Evaluate Biocompatibility through Measuring the Hemolysis Rate

Conducting hemolysis rate testing is a crucial step in assessing the blood compatibility of AuNC·Gd–Cy7 nanoparticles and Sur–AuNC·Gd–Cy7 nanoprobes, as it will provide valuable insights into their potential for in vivo use.

Blood samples (6 mL) from healthy rats were collected in 10-milliliter heparin-coated tubes. The blood sample was purified by centrifugation to remove serum at 1500 rpm for 5 min, and the precipitate of red blood cells (RBCs) was washed with sterile physiological saline solution (0.9% NaCl) on a centrifugal machine three times. The precipitate of RBCs was re-suspended in sterile physiological saline solution, while the supernatant was not red colored. Then AuNC·Gd nanoparticles (50 μL, 1 μg/mL), Sur–AuNC·Gd (50 μL, 1 μg/mL), and Sur–AuNC·Gd–Cy7 nanoprobes (50 μL, 100 μL, and 1 μg/mL) were mixed with the suspension solution, respectively.

Negative control and positive control were obtained by mixing up 3 mL of suspension of RBCs with 2 mL of physiological saline solution (0.9% NaCl) and 2 mL of pure water, respectively.

After incubation for 3 h, the photograph was taken in order to show the hemolytic reaction of all the samples. Then the supernatant of each sample was gathered overnight. The absorbance values of the supernatant were measured at 540 nm on a UV-visible spectrophotometer (UV-3600, Shimadzu, Japan). The hemolysis rate (HR) was tested and calculated by the following equation:

HR = (OD_Au_ − OD_C1_) × 100%/(OD_C2_ − OD_C1_) (1)

In this equation, OD_Au_ is the absorbance value of the supernatant of nanoparticles and the nanoprobe groups. OD_C1_ and OD_C2_ are the absorbance values of the negative control group and the positive control, respectively.

### 2.11. The Establishment of a Nude Mouse Orthotopic Pancreatic Cancer Model

The nude mice (5–6 weeks old) of clean grade were fed in the specific-pathogen-free (SPF) animal center of Nanjing University of Traditional Chinese Medicine. This study was approved by the Animal Ethics Committee of the Affiliated Hospital of Nanjing University of Traditional Chinese Medicine. All experimental protocols were reviewed by the Animal Ethics Committee of the Affiliated Hospital of Nanjing University of Traditional Chinese Medicine.

The green fluorescent protein (GFP)-transfected pancreatic cancer cell line BxPC-3 was cultured at 37 °C in a humidified 5% CO_2_ atmosphere. The cells were injected subcutaneously into the abdominals of nude mice while they were in the logarithmic phase. The subcutaneous tumor model was constructed first. When the maximum diameter of the subcutaneous tumor was up to 8–10 mm, it was dissected and cut into small fragmented tissues (about 1 mm × 1 mm in size) for backup. Take the healthy nude mouse; its pancreas was exposed by operation. Small fragments of tissue were sutured into the pancreas to build a nude mouse orthotopic pancreatic cancer model. The mice were fixed position after anesthesia and placed under an animal fluorescence system to observe the growth and size of the orthotopic tumor seven days after surgery. The nude mice were ready for future in vivo experiments, while the diameter of the tumor was about 5 mm.

### 2.12. In Vivo MR and Fluorescence Imaging of Tumors

Nude mice with orthotopic pancreatic tumors were fed in an SPF environment. Animal weight and tumor size were monitored. Approximately when the tumor volume reached about 100 mm^3^, in vivo MR imaging was performed on a clinical 3T MR imaging scanner (SIEMENS Verio 3.0 T) to observe the targeting accumulation of target Sur–AuNC·Gd–Cy7 nanoprobes and non-target AuNC·Gd–Cy7 nanoprobes in tumors. The nanoprobes (1 mg [Gd]/kg) were injected via the tail vein.

Then the MR imaging was performed pre-injection and at different time points (12 h, 24 h, 48 h, and 72 h) after the injections. The T1 scanning parameters were as follows: TR = 490.0 ms, TE = 14.0 ms, flip angle = 180.0°, averages = 1, FOV = 4 cm × 4 cm, matrix = 256 × 256, slice thickness = 0.8 mm. Axial and coronal T1WI scans were performed, respectively. An ROI of 0.5 × 0.5 cm^2^ was circled, and the T1 values were measured and plotted, respectively.

Approximately when the tumor volume reached about 100 mm^3^, in vivo near-infrared fluorescence imaging was performed on a small animal vivisection imaging system (GE). A targeted Sur–AuNC·Gd–Cy7 nanoprobe was injected via the tail vein. The in vivo NIR imagers were recorded before, 12 h, 24 h, 48 h, and 72 h after injection. The fluorescence image of the tumor-bearing GFP was taken at the excitation wavelength and emission wavelength of 488 nm and 507 nm, respectively. The target effect of the Sur–AuNC·Gd–Cy7 nanoprobe was examined at the excitation wavelength and emission wavelength of 750 nm and 788 nm, respectively. Pseudo-color was added to each image to analyze the targeting in vivo. At the endpoint of imaging, the corresponding organs (tumor, liver, lung, spleen, kidney, and heart) of nude mice with tumors were taken out for imaging ex vivo and future applications.

The organs (tumor, liver, lung, spleen, kidney, and heart) and tumors harvested from mice were subjected to histopathological examination. Hematoxylin and eosin (HE) staining was used to evaluate the pathological feature. Typically, organs and tumor tissues were fixed in 10% formalin for about 4 h before processing into paraffin. The blocks were sliced into micrometer-thick slices and stained with hematoxylin and eosin (HE). The morphology of tissue cells was examined with an optical microscope.

### 2.13. The Content of Nanoprobes in Tissues

Six-week-old nude mice were adopted for the analysis of Au content in organs. The mice (3 injected with Sur–AuNC·Gd–Cy7 and 3 injected with AuNC·Gd–Cy7) were euthanized at 12 h, 24 h, and 48 h post-injection. The organs (liver and kidney) and the blood were harvested for the content of the Au analysis. They were weighed accurately. Then they were cut into pieces and dissolved in aqua regia overnight. Analysis of Au content was determined by ICP-AES. Moreover, three mice without the injection of nanoprobes were used as controls.

### 2.14. Statistical Analysis

All data are presented by mean ± standard deviation (SD). The data was statistically managed and compared with variance analysis (two-way ANOVA, one-way ANOVA, and *t*-test). The survival curve was performed using the Kaplan–Meier method. A difference with *p* < 0.05 was considered statistically significant. Then, the data was set at *p* < 0.05 (*), *p* < 0.01 (**), and *p* < 0.001 (***), respectively.

## 3. Results

### 3.1. Synthesis and Characterization of Nanocages and Nanoprobes

**Figure 1:**
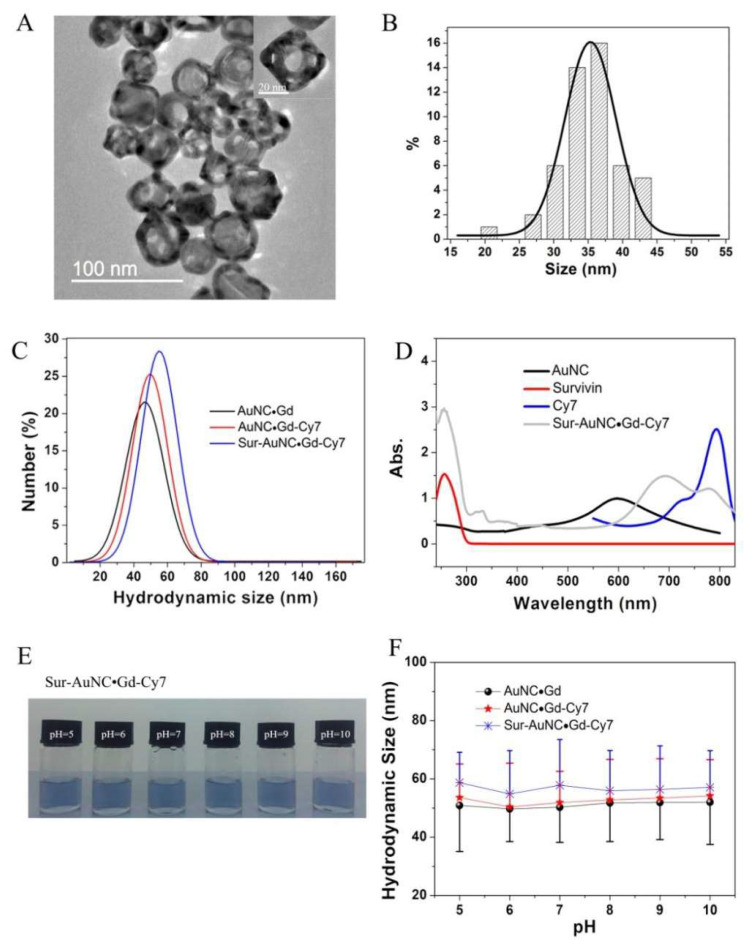
(**A**,**B**) TEM imaging (**A**) and the statistical sizes (**B**) of AuNC nanoparticles. (**C**) Dynamic light scattering diameter distributions of AuNC•Gd, AuNC•Gd–Cy7 particles, and Sur–AuNC•Gd–Cy7 nanoprobes. (**D**) UV-vis absorption spectroscopy curves of AuNC, survivin, Cy7, and Sur–AuNC•Gd–Cy7 nanoprobes. (**E**) Stability images of Sur–AuNC•Gd–Cy7 nanoprobes in aqueous solutions with different pHs. (**F**) Stability curves of various AuNC•Gd, AuNC•Gd–Cy7 particles, and Sur–AuNC•Gd–Cy7 nanoprobes in aqueous solutions with different pHs.

We synthesized Au nanocages and survivin-modified AuNC loading fluorescence of Cy7 and T1 contrast agent Gd. The synthesis process of Sur–AuNC·Gd–Cy7 nanoprobes is shown in Scheme 1A. The representative TEM images of the prepared AuNC, together with their corresponding particle size distribution profiles, are shown in Figure 1A,B. As can be seen from the figures, average sizes of 35.3 nm with a uniform hollow, porous structure were obtained. The hydrodynamic sizes of AuNC, AuNC•Gd–Cy7 nanoparticles, and Sur–AuNC•Gd–Cy7 nanoprobes were obtained by Dynamic Light Scattering. They are 50.16 ± 15.83 nm, 52.48 ± 15.98 nm, and 55.78 ± 16.47 nm, respectively (Figure 1C). The zeta potentials are -12.6 ± 6.26 mV, −15.4 ± 9.33 mV, and −14.3 ± 11.1 mV, respectively (Figure S1D). The UV-vis absorption spectroscopy curves of AuNC, survivin, Cy7, and Sur–AuNC•Gd–Cy7 nanoprobes are shown in Figure 1D. The curve of Sur–AuNC•Gd–Cy7 contains the peak of AuNC, survivin, and Cy7, which indicates that the nanoprobes were synthesized successfully. The Sur–AuNC•Gd–Cy7 nanoprobes in an aqueous solution were stable, and the hydrodynamic sizes of AuNC•Gd, AuNC•Gd–Cy7 particles, and Sur–AuNC•Gd–Cy7 nanoprobes changed a little in different pH conditions (Figure 1E,F).

**Figure 2:**
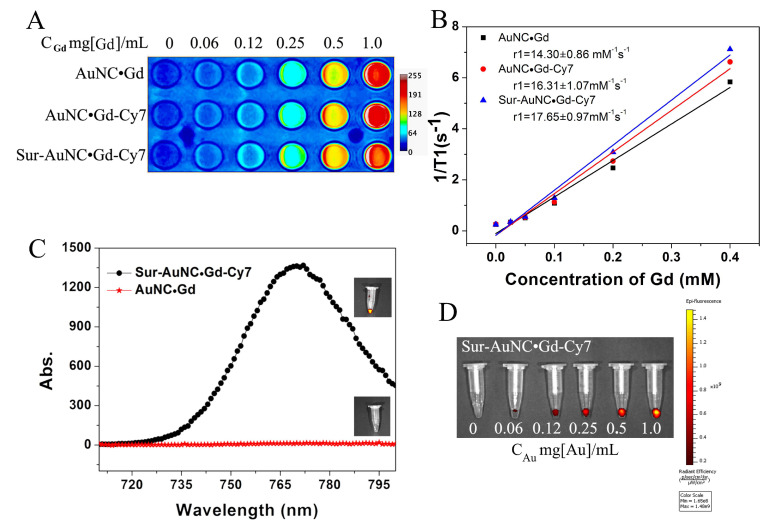
(**A**) T1−weighted MR images of AuNC•Gd, AuNC•Gd−–Cy7 particles, and Sur–AuNC•Gd−Cy7 nanoprobes. (**B**) Water proton longitudinal relaxation rate (1/T1) of AuNC•Gd, AuNC•Gd−Cy7 particles, and Sur−AuNC•Gd−Cy7 nanoprobes in aqueous solution as a function of Gd concentration. (**C**) Fluorescence spectra curves of AuNC•Gd particles and Sur−AuNC•Gd−Cy7 nanoprobes. (**D**) Fluorescence images of Sur−AuNC•Gd−Cy7 nanoprobes with different concentrations.

The imaging characteristics of AuNC•Gd, AuNC•Gd–Cy7 particles, and Sur–AuNC•Gd–Cy7 nanoprobe solutions were tested on a 3.0T clinical magnetic resonance imaging and fluorescent spectrometer. With the concentration of Gd increasing, the signal of T1-weighted images has a high enhancement (Figure 2A). The following Figure 2B illustrates the corresponding magnetic relaxation rate based on gadolinium concentration (r1_AuNC•Gd_ = 14.30 ± 0.86 mM^−1^s^−1^, r1 _AuNC•Gd–Cy7_ = 16.31 ± 1.07 mM^−1^s^−1^, r1 _Sur–AuNC•Gd–Cy7_ = 17.65 ± 0.97 mM^−1^s^−1^), which is much higher than that of magnevist (r1 = 4.5 mM^−1^s^−1^) [18]. The Sur–AuNC•Gd–Cy7 nanoprobes solution has good fluorescence imaging performance (Figure 2C,D). The Sur–AuNC•Gd–Cy7 nanoprobes have the ability to generate fluorescence images after loading with Cy7. The nanoprobes displayed a strong fluorescence signal when excited at 750 nm. The nanoprobes had a high signal-to-background ratio, and the fluorescence intensity was stable over a wide range of concentrations.

### 3.2. Precise Targeting

**Figure 3:**
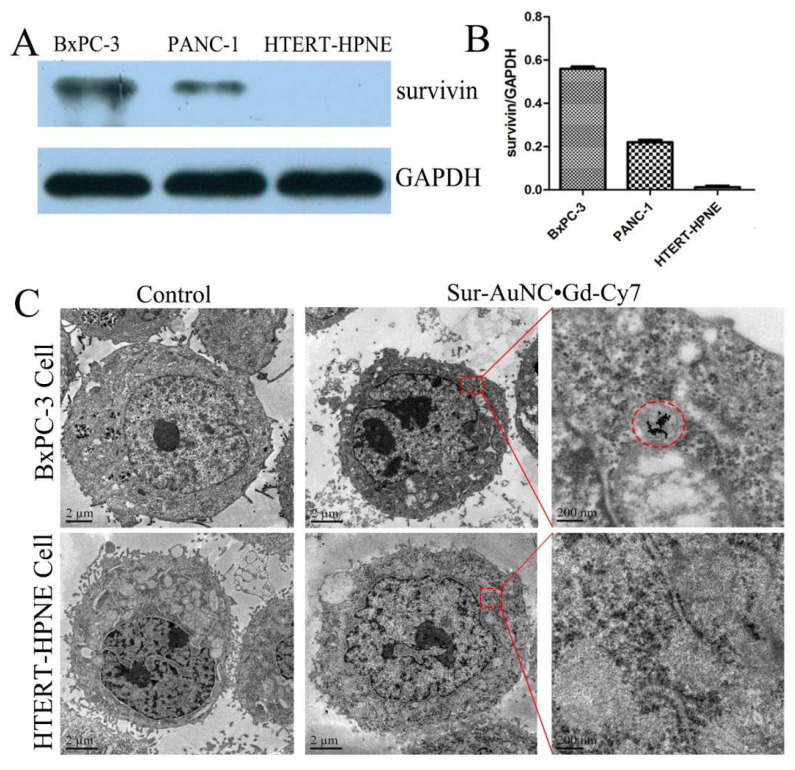
(**A**,**B**) Western blotting test (**A**) of survivin protein expression in different cell lines and their relative contents (**B**). (**C**) Bio-TEM images of the structural evolution of Sur–AuNC•Gd–Cy7 nanoprobes in BxPC-3 cancer cells and HTERT-HPNE cells after 24 h of incubation and their enlarged view. The red dashed circles contain black dots which signify nanoprobes that have been taken into the cell.

Distinct levels of survivin protein are observed in pancreatic cancer cells (Figure 3A,B). The human pancreatic ductal epithelial cells HTERT-HPNE do not naturally express survivin protein. The level of survivin protein expressed in BxPC-3 cells is higher compared to that expressed in PANC-1 cells. Vesicles containing nanoprobes are seen in the BxPC-3 cells after incubation with Sur–AuNC•Gd–Cy7 nanoprobes for 24 h (Figure 3C). As a control, no probe vesicles are seen in the HTERT-HPNE cells. Part of the reason is that pancreatic cancer BxPC-3 cells have a vigorous metabolism. Notably, the survivin protein is absent in the HTERT-HPNE cells, while the BxPC-3 cells demonstrate a marked presence of the survivin protein.

**Figure 4:**
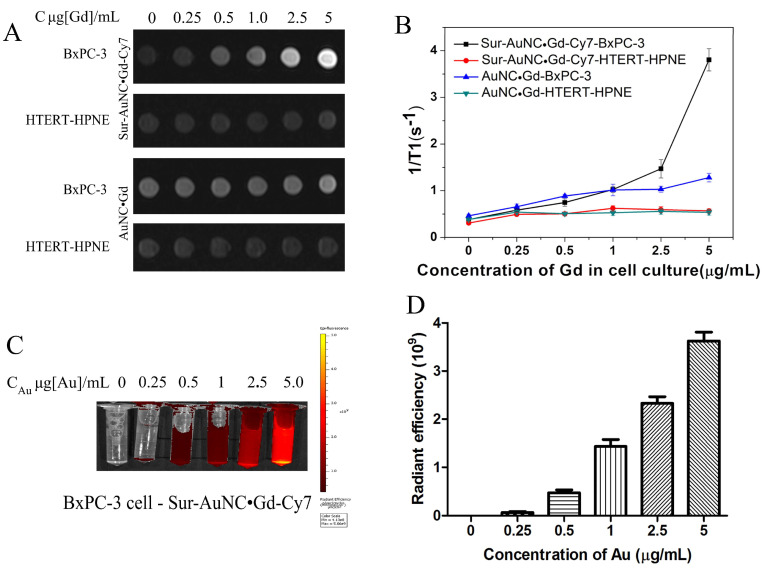
(**A**,**B**) MR signal images (**A**) and MR signal intensity (**B**) of different cell lines after incubation with AuNC•Gd particles and Sur–AuNC•Gd–Cy7 nanoprobes for 24 h. (**C**,**D**) Fluorescence signal image (**C**) and signal changes (**D**) of BxPC-3 cells after incubation with different concentrations of Sur–AuNC•Gd–Cy7 nanoprobes for 24 h.

After being exposed to different concentrations of AuNC•Gd particles and Sur–AuNC•Gd–Cy7 nanoprobes for 24 h, BxPC-3 cells and HTERT-HPNE cells were harvested and dispersed on agarose solutions. The T1 signal intensity was detected (Figure 4A,B). Evidently, the T1 signal intensity of BxPC-3 cells that were subjected to the targeted Sur–AuNC•Gd–Cy7 nanoprobes was considerably greater than that of cells that were subjected to the AuNC•Gd particles. The outcome is in line with the findings of cellular electron microscopy. Upon exposure to the Sur–AuNC•Gd–Cy7 nanoprobes, the T1 signal intensity exhibited by HTERT-HPNE cells was observed to be comparatively lower than that of BxPC-3 cells. The fluorescence signal exhibited by the Sur–AuNC•Gd–Cy7 nanoprobes remained constant even after being subjected to incubation with BxPC-3 cells (Figure 4C,D). The results of the experiment demonstrate that the targeted Sur–AuNC•Gd–Cy7 nanoprobes are effective at targeting pancreatic cancer cells. The findings also suggest that the nanoprobes could be used as potential imaging reagent.

### 3.3. Pro-Apoptotic Studies on Pancreatic Cells

**Figure 5:**
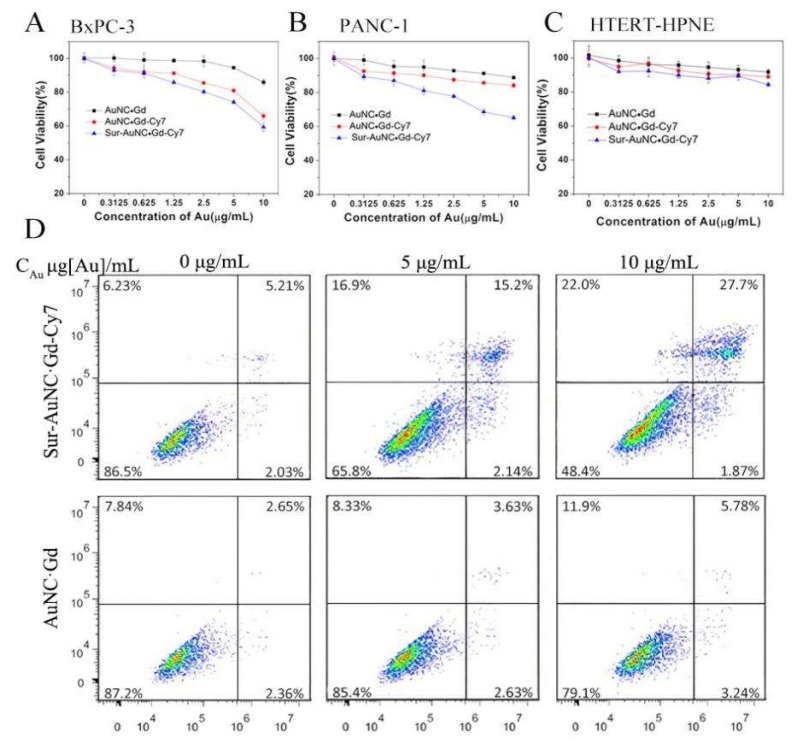
(**A**–**C**) The cytotoxicity performance of AuNC•Gd, AuNC•Gd–Cy7 particles, and Sur–AuNC•Gd–Cy7 nanoprobes in BxPC-3 cells (**A**), PANC-1 cells (**B**), and HTERT-HPNE cells (**C**) after incubation for 24 h. (**D**) Flow cytometric analyses of BxPC-3 cells after incubation with AuNC•Gd particles and Sur–AuNC•Gd–Cy7 nanoprobes for 24 h.

As survivin proteins are known to impede apoptosis, it is plausible that directing therapeutic efforts toward these proteins could facilitate apoptosis. It is evident that the proliferation of BxPC-3 and PANC-1 cells exhibiting high protein expression is effectively suppressed by the presence of high concentrations of the Sur–AuNC•Gd–Cy7 nanoprobes (Figure 5A,B). Due to the absence of survivin protein expression in the HTERT-HPNE cells, there was no hindrance to their proliferation (Figure 5C). The flow cytometry apoptosis outcomes observed in HTERT-HPNE cells have validated the aforementioned claim (Figure S3). The proliferation of BxPC-3 cells was effectively inhibited by Sur–AuNC•Gd–Cy7 nanoprobes through the utilization of the pro-apoptotic pathway (Figure 5D). The uncomplicated AuNC•Gd particles and AuNC•Gd–Cy7 particles, which did not possess any targeting properties, were unable to induce apoptosis in BxPC-3 cells and demonstrated no inhibitory impact on the proliferation of BxPC-3, PANC-1, and HTERT-HPNE cells.

### 3.4. In Vivo Fluorescence Imaging

Verification of the biocompatibility of the AuNC•Gd, AuNC•Gd–Cy7 particles, and Sur–AuNC•Gd–Cy7 nanoprobes was achieved by analyzing hemolysis rates. (Figure S4). Particles and nanoprobes exhibited a hemolysis percentage of under 5%. This result indicates that these particles and nanoprobes are non-toxic to red blood cells and can be used safely in biomedical applications.

**Figure 6:**
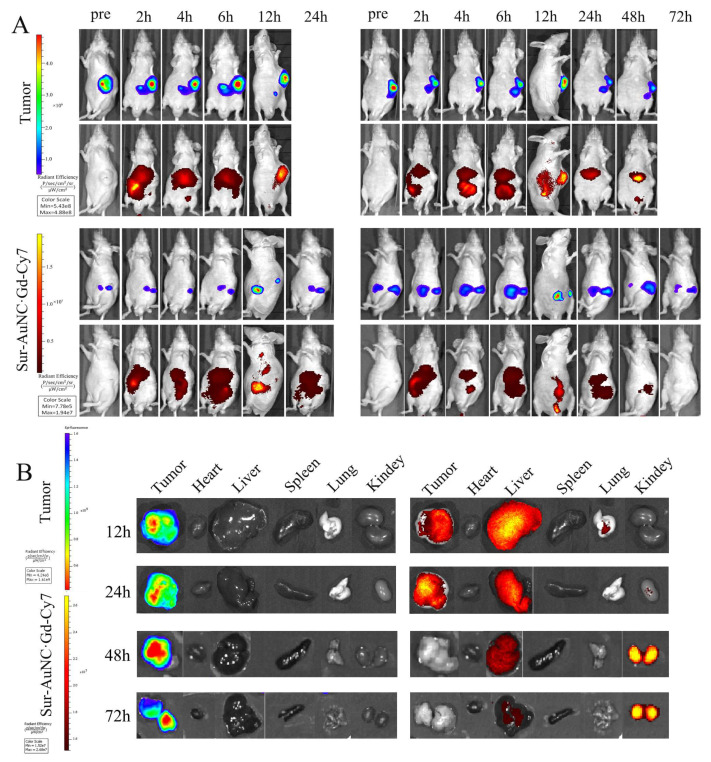
(**A**) Fluorescence images of BxPC-3 xenograft-bearing mice after intravenous (i.v.) injection of Sur–AuNC•Gd–Cy7 nanoprobes at different times. (**B**) Ex vivo images of major organs, such as the heart, liver, spleen, lung, kidney, and tumors excised at different times post-injection.

To evaluate the fluorescence imaging efficiency of the Sur–AuNC•Gd–Cy7 nanoprobes in vivo, the BxPC-3 tumor in situ model was established on nude mice, and the contrast agent AuNC•Gd–Cy7 particles and Sur–AuNC•Gd–Cy7 nanoprobes were injected via tail vein (Scheme 1B). As shown in Figure 6A and S5, a positive targeted imaging outcome is observed in the BxPC-3 tumor when utilizing the Sur–AuNC•Gd–Cy7 nanoprobes. The fluorescence signal in the tumor sites was highest at 12 h and 24 h postinjection for Sur–AuNC•Gd–Cy7 nanoprobes. The AuNC•Gd–Cy7 particles had no targeted fluorescence imaging effect in BxPC-3 tumors. The biodistribution of the Sur–AuNC•Gd–Cy7 nanoprobes and AuNC•Gd–Cy7 particles was further investigated by ex vivo imaging. As shown in Figure 6B and S5B, the fluorescence signal of Sur–AuNC•Gd–Cy7 nanoprobes in the tumor sites was strong at 12 and 24 h. After 48 h, the renal fluorescence signal exhibited a marked increase, indicating renal metabolism of the Sur–AuNC•Gd–Cy7 nanoprobes and AuNC•Gd–Cy7 particles. After 72 h, the liver’s fluorescence intensity decreased, suggesting that the Sur–AuNC•Gd–Cy7 nanoprobes may be eliminated from the liver through metabolic processes within a defined duration. The results of this study suggest that the Sur–AuNC•Gd–Cy7 nanoprobes had a higher tendency to accumulate at the tumor sites in comparison to the AuNC•Gd particles.

### 3.5. In Vivo MR Imaging and Pathological Examination

**Figure 7:**
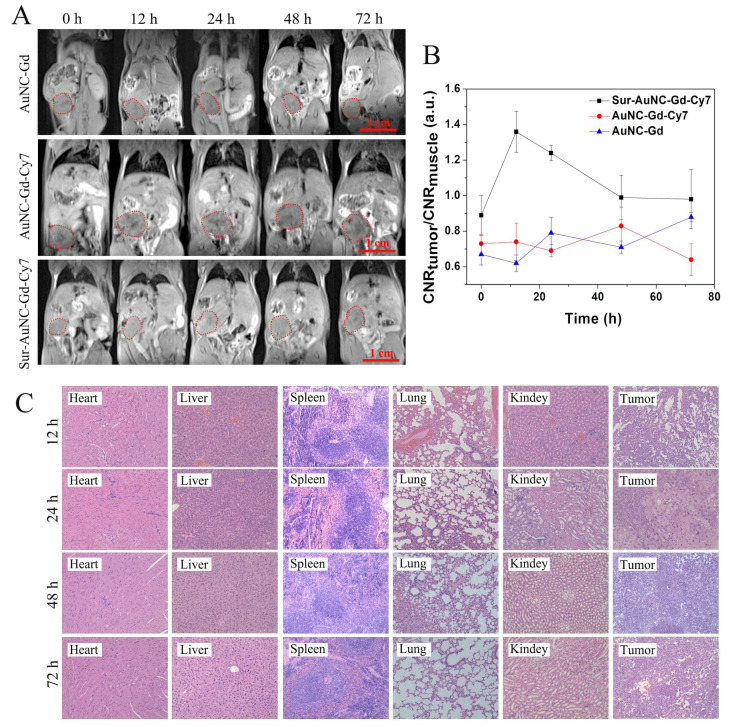
(**A**) T1-weighted tumor contrast enhancement after i.v. injection of AuNC•Gd, AuNC•Gd–Cy7 nanoparticles, and Sur–AuNC•Gd–Cy7 nanoprobes. (**B**) The changes in MR signal intensity of tumor tissue after treatment with nanoparticles and nanoprobes. (**C**) H&E analyses of different organ tissues and tumor tissues after treatment with Sur–AuNC•Gd–Cy7 nanoprobes, magnification 100×.

The utilization of multimodal imaging evaluation could prove advantageous in the timely identification of pancreatic ductal adenocarcinoma (PDAC) and image-directed treatment of the malignancy. Following the administration of active-targeted Sur–AuNC•Gd–Cy7 nanoprobes, the T1WI MR signal intensity at the orthotopic tumor sites exhibited a pattern that was reliant on time. It attained its maximum value at the 12-h mark and subsequently declined gradually, as demonstrated in Figure 7A. In the absence of a targeting protein modification, the signal of the AuNC•Gd and AuNC•Gd–Cy7 particles within the tumor remained inconspicuous (Figure 7A). After conducting a semiquantitative analysis, it was found that the T1 value at the tumor site was considerably higher at the 12-h mark following the administration of Sur–AuNC•Gd–Cy7 nanoprobes in comparison to both untargeted AuNC•Gd and AuNC•Gd–Cy7 particles (Figure 7B). The T1 signal of the Sur–AuNC•Gd–Cy7 nanoprobes demonstrated a decrease within the tumor after 48 h, consistent with the fluorescence outcomes. The findings indicate that the Sur–AuNC•Gd–Cy7 nanoprobes, which employ active targeting, exhibit an increased propensity for tumor aggregation as a result of their specific binding abilities as compared to their passive-targeting counterparts.

After conducting HE stains, it was found that there were no significant abnormalities in the hearts, livers, spleens, lungs, or kidneys. This provides additional proof that the administration of imaging doses has an exceptional safety profile in vivo, as depicted in Figure 7C. The pro-apoptotic effect of the Sur–AuNC•Gd–Cy7 nanoprobes on tumor cells was not evident based solely on the HE staining results (100×), possibly due to the limited duration of exposure.

## 4. Discussion

The development of pancreatic cancer is a multifaceted and ongoing pathological process that entails the dysregulation of numerous proteins, genes, and enzymes. This includes the activation and deactivation of various oncogenes and tumor suppressor genes, as well as the promotion and inhibition of apoptotic and anti-apoptotic genes [19]. Survivin, a member of the inhibitor of an apoptosis protein family, is a small protein that is highly expressed in tumors [20]. Its overexpression is frequently associated with poor prognoses in several human neoplasms. This inhibitor of apoptosis plays a crucial role in promoting cancer cell survival while simultaneously inhibiting cell death [21].

Survivin serves multiple purposes, including the inhibition of cell apoptosis, regulation of the cell cycle, promotion of cell proliferation, stimulation of cell mitosis, and enhancement of vascular proliferation. The protein possesses a coiled-like structure at its C-terminal that collaborates with the division apparatus to modulate the process of cell mitosis. Additionally, the protein’s N-terminal BIR structure can impede cell apoptosis by interacting with caspases in a direct or indirect manner [20,22]. Survivin is known to exhibit high expression in the majority of malignant tumor tissues while remaining absent in normal tissues, thereby indicating its specificity towards tumors to a significant degree. This characteristic has led to the utilization of survivin as a specific target for the treatment of tumors [21,23].

The cellular specificity of survivin results in its differential distribution among tissues. According to reports, normal pancreatic tissues and those affected by chronic pancreatitis do not express the survivin gene. Conversely, pancreatic cancer tissues exhibit a high survivin expression rate of 76.9%, and Bor peptide has been shown to have a promising future in cancer imaging and anti-cancer drugs that target survivin, according to studies [24]. Survivin has emerged as a highly promising target for the development of targeted therapies for pancreatic cancer, with the potential to enable early diagnosis and precise treatment of the disease [25]. Survivin-targeting nano-delivery systems have been utilized to successfully inhibit the growth of tumors [23]. The Sur–AuNC•Gd–Cy7 nanoprobe that we have developed, which targets survivin for the purpose of diagnostic imaging of pancreatic cancer, is founded on both theoretical and experimental grounds.

The remarkable properties of gold nanocages, including their special hollow and porous structure, large absorption cross-section, and favorable attributes such as low toxicity, good biocompatibility, and ease of surface modification, have made them a prominent focus of investigation in the field of nanomedicine [17,26]. AuNCs carriers have the potential to induce both endogenous and exogenous responses for the diagnosis and treatment of tumors. This implies that they can be used to facilitate the identification and resolution of tumors through natural mechanisms, and this process can also be affected by employing external stimuli, such as temperature, light, or ultrasound. Additionally, AuNC-based nanocarriers that exhibit sensitivity to dual or multiple stimuli have been studied to enhance their adaptability to the cancer cell microenvironment and increase their specificity and effectiveness [17,26]. The use of a multimodal stimuli imaging evaluation has the potential to be advantageous in the timely detection of PDAC and the application of image-directed treatment for malignancy [27]. Consequently, our Sur–AuNC•Gd–Cy7 nanoprobe utilizes a dual-response imaging approach that combines magnetic resonance and fluorescence.

In previous studies, we successfully constructed a dual-modality molecular probe that targets pancreatic cancer by utilizing chitosan-encapsulated magnetic nanoparticles as a carrier and targeting the survivin gene. The results of magnetic resonance imaging have shown a considerable reduction in the T2 signal of the tumor tissue, while Prussian blue staining has confirmed the specific distribution of the molecular probe within pancreatic cancer tissue, thus facilitating early targeted molecular imaging [8,28]. The current investigation utilized near-infrared fluorescence and magnetic resonance modalities for in vivo imaging and subsequently contrasted the imaging results with pathological observations to explore the targeted accumulation effect of Sur–AuNC•Gd–Cy7 nanoprobe on pancreatic cancer in vivo. While still utilizing the survivin gene, the nanoprobes developed in this study display satisfactory biocompatibility and targeting efficacy.

The findings demonstrated that the AuNC•Gd–Cy7 and AuNC•Gd–Cy7 nanoparticles, as well as the Sur–AuNC•Gd–Cy7 nanoprobe, exhibited hemolysis rates below 5%. This level of hemolysis rate meets the medical biomaterial standard, signifying that the nanoprobe and nanoparticles did not cause any harm to red blood cells and had favorable blood compatibility. The results of near-infrared fluorescence imaging indicated that the probe’s fluorescence was detectable in the majority of the tumor area 12 h post-injection. Furthermore, a marginal increase in signal intensity on T1WI confirmed the probe’s effective targeting of pancreatic cancer tissue. According to the pathological HE staining, there were no noticeable alterations in the cellular morphology of the tumor, nor were there any significant modifications in the structure of the organs. Thus, the Sur–AuNC•Gd–Cy7 probe exhibits remarkable efficacy in targeting the accumulated tissue of pancreatic cancer tumors.

To achieve a more effective diagnostic result, a multitude of challenges must still be addressed. One instance is the issue of signal amplification, which involves determining the most suitable imaging duration and concentration of targeted probes. The growth rate of tumors can become too rapid after formation, thereby reducing the diagnostic precision of MR imaging as the tumor tissue expands, ultimately impacting the analysis of experimental outcomes. The forthcoming studies will involve the application of AuNCs in photothermal therapy to exploit the carriers’ inherent features in the detection and treatment of pancreatic cancer.

## 5. Conclusions

In the present study, we have accomplished the construction of a multi-modal molecular nanoprobe, Sur–AuNC•Gd–Cy7, that specifically targets the survivin gene. Modifying PEG can effectively reduce the cytotoxicity of nanoprobes, and incorporating survivin modifications can significantly improve their targeting efficiency. The Sur–AuNC•Gd–Cy7 nanoprobe exhibits a high degree of efficacy in targeting pancreatic cancer BxPC-3 cells, and at a low concentration, it is capable of inducing T1WI signal enhancement. Cell electron microscopy analysis revealed that the Sur–AuNC•Gd–Cy7 probe was present in the cytoplasm of BxPC-3 cells, indicating that the probe possesses a strong targeting capacity towards pancreatic cancer cells in vitro. This pancreatic cancer-targeted nanoprobe, Sur–AuNC•Gd–Cy7, has been shown to selectively accumulate in the tumor area. The probe’s fluorescence is observable in the tumor tissue, and the signal intensity of the tumor region is slightly increased on T1WI, indicating its potential as a diagnostic tool. Through the use of in vivo imaging, it has been observed that the nanoprobe Sur–AuNC•Gd–Cy7 possesses the ability to specifically target pancreatic cancer. The results indicate a favorable in vivo targeting effect of the Sur–AuNC•Gd–Cy7 nanoprobe, thereby establishing a solid experimental foundation for the application of clinical molecular imaging in pancreatic cancer.

## Data Availability

All data is uploaded and available to readers.

## Supplementary Materials

The following supporting information can be downloaded at: https://www.mdpi.com/article/10.3390/pharmaceutics15051547/s1, Figure S1: Hydrodynamic size of AuNC•Gd, AuNC•Gd–Cy7 nanoparticles, and Sur–AuNC•Gd–Cy7 nanoprobes at intensity (A) and PDI respectively (B). The TEM image (C) of AuNC•Gd, AuNC•Gd–Cy7 nanoparticles, and Sur–AuNC•Gd–Cy7 nanoprobes shows zeta potential, respectively (D). Figure S2: The Au concentrations of AuNC•Gd–Cy7 nanoparticles and Sur–AuNC•Gd–Cy7 nanoprobes in tumors and organs at different time points after i.v. injection via the tail vein. Figure S3: The hemolysis experiment (A) and calculated hemolysis (B) of AuNC•Gd, AuNC•Gd–Cy7 nanoparticles, and Sur–AuNC•Gd–Cy7 nanoprobes. Figure S3. Flow cytometric analyses of HTERT-HPNE cells after incubation with AuNC•Gd nanoparticles and Sur–AuNC•Gd–Cy7 nanoprobes for 24 h. Figure S4. (A) Fluorescence images of BxPC-3 xenograft-bearing mice after intravenous (i.v.) injection of AuNC•Gd–Cy7 nanoparticles at different times. (B) Ex vivo images of major organs, such as the heart, liver, spleen, lung, kidney, and tumors excised at 72 h post-injection.
