# Supplementary material for: A Gold Nanocage Probe Targeting Survivin for the Diagnosis of Pancreatic Cancer
Source: Pharmaceutics. 2023 May 19;15(5):1547. doi: 10.3390/pharmaceutics15051547 (PMC10221143; doi:10.3390/pharmaceutics15051547)
Supplement: Supplementary file 1 [file pharmaceutics-15-01547-s001.zip › pharmaceutics-2368708-supplementary.pdf]

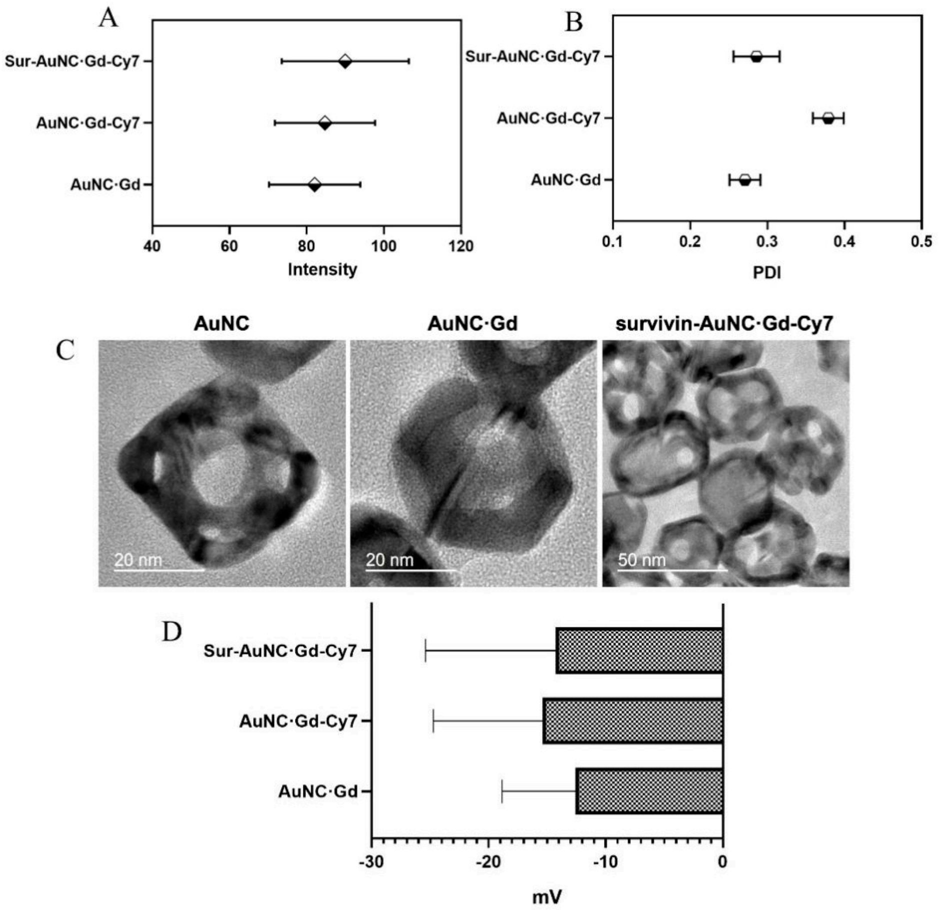

Figure S1. Hydrodynamic size of AuNC•Gd, AuNC•Gd-Cy7 nanoparticles and Sur-AuNC•Gd-Cy7 nanoprobes at Intensity(A) and relatively PDI (B). The TEM image (C) of AuNC•Gd, AuNC•Gd-Cy7 nanoparticles and Sur-AuNC•Gd-Cy7 nanoprobes and relatively zeta potential (D).

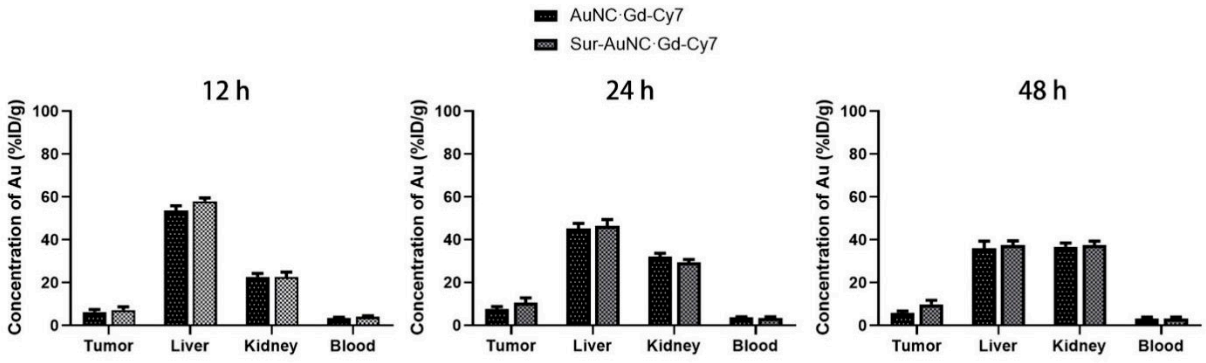

Figure S2. The Au concentrations of AuNC•Gd-Cy7nanoparticles and Sur-AuNC•Gd-Cy7 nanoprobes in tumors and organs at different time points after iv vie tail vein.

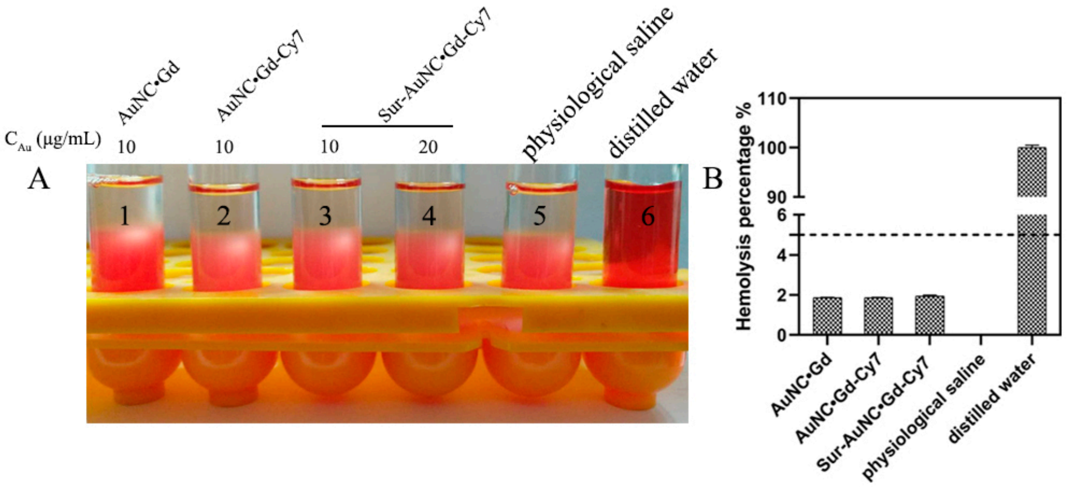

Figure S3. The haemolysis experiment (A) and calculated haemolysis (B) of AuNC•Gd, AuNC•Gd-Cy7 nanoparticles and Sur-AuNC•Gd-Cy7 nanoprobes.

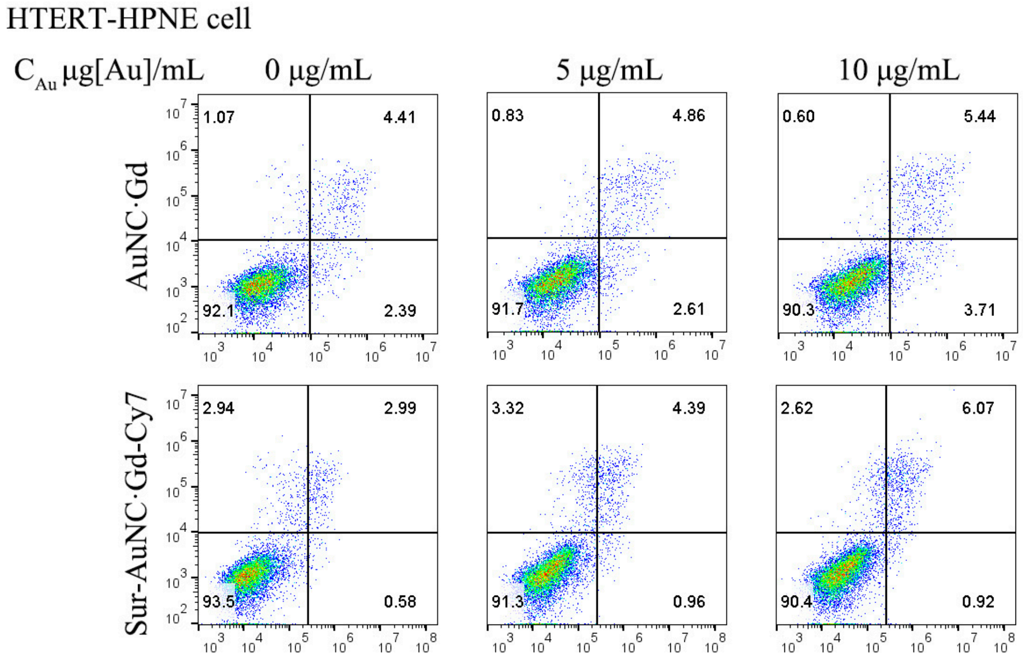

Figure S4. Flow cytometric analyses of HTERT-HPNE cells after incubation with AuNC•Gd nanoparticles and Sur-AuNC•Gd-Cy7 nanoprobes for 24h.

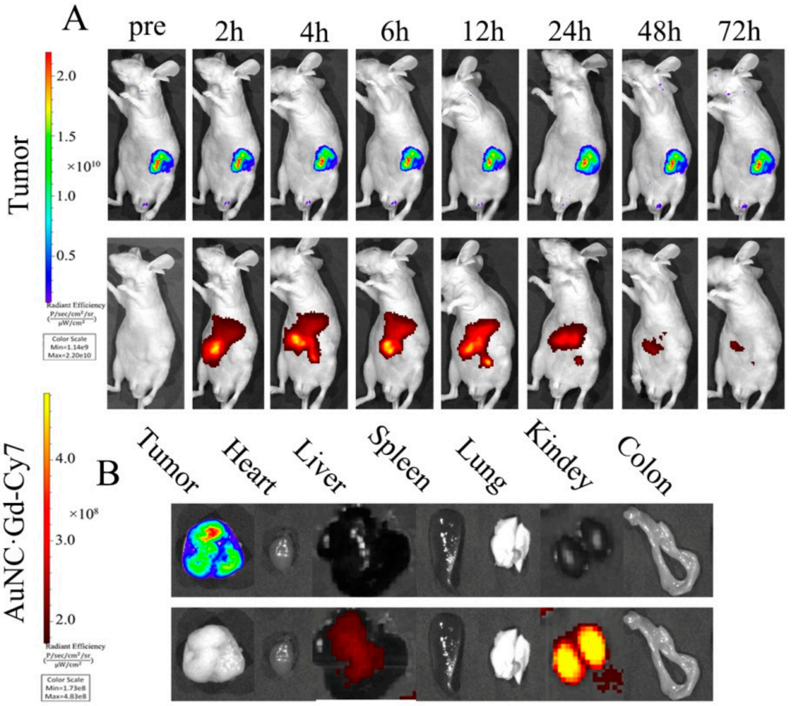

Figure S5. (A) Fluorescence images of BxPC-3 xenograft-bearing mice after intravenous (i.v.) injection of AuNC•Gd-Cy7 nanoparticles at different times. (B) Ex vivo images of major organs, such as heart, liver, spleen, lung, kidney, and tumors excised at 72h post-injection.
